# Complete Chloroplast Genome of *Nepeta griffithii* and Comparative Genomics of 10 *Nepeta* Species

**DOI:** 10.1002/ece3.74582

**Published:** 2026-10-25

**Authors:** Xinyi Li, Abdul Sammad, Sayed Afzal Shah, Jiahui Sun, Aman Karim, Bartholomew Yir‐erong, Xiaoxuan Tian, Abdullah

**Affiliations:** ^1^ State Key Laboratory of Chinese Medicine Modernization Tianjin University of Traditional Chinese Medicine Tianjin China; ^2^ Haihe Laboratory of Modern Chinese Medicine Tianjin China; ^3^ Department of Biotechnology National University of Medical Sciences Rawalpindi Pakistan; ^4^ State Key Laboratory for Quality Ensurance and Sustainable Use of Dao‐di Herbs, National Resource Center for Chinese Materia Medica China Academy of Chinese Medical Sciences Beijing P. R. China; ^5^ Institute of Traditional and Alternative Medicine (ITAM) University of Health and Allied Sciences (UHAS) Ho Ghana

**Keywords:** chloroplast genome, comparative genomics, Lamiaceae, *Nepeta griffithii*, nucleotide diversity, phylogeny

## Abstract

*Nepeta* L. (Lamiaceae) is an ecologically and medicinally important genus, yet complete cp genome resources are available for fewer than 10 of the approximately 300 accepted species, with western Himalayan taxa almost entirely unrepresented. Here, we sequenced, assembled, and annotated the complete cp genome of *Nepeta griffithii* and compared it with nine previously published *Nepeta* cp genomes. The 10 cp genomes exhibited a highly conserved quadripartite structure, with genome sizes ranging from 151,254 to 152,348 bp, and contained 113 unique genes (79 protein‐coding, 30 tRNA, 4 rRNA). Gene content, guanine‐cytosine content, intron features, and inverted repeat contraction and expansion were highly conserved. SSR analysis identified 387 repeats across the 10 cp genomes, predominantly mononucleotide A/T motifs in intergenic spacers, yielding candidate cpSSR markers for population‐level studies. Codon usage analysis revealed a strong bias toward A/T‐ending codons, with leucine as the most frequent amino acid. Nucleotide diversity analysis showed intergenic spacers were more variable than both introns and genic regions. Highly polymorphic loci, including *ccsA‐ndhD*, *rpl32‐trnL‐UAG*, and *ndhG‐ndhI*, were identified as candidate markers for phylogenetic and population studies. Maximum likelihood phylogeny based on 79 concatenated protein‐coding genes recovered a well‐resolved topology within *Nepeta* and placed *N. griffithii* as sister to *N. nuda* with maximal bootstrap support of 100%. This study provides the first complete cp genome for *N. griffithii*, improves plastome sampling within the genus, and offers genomic markers for species identification, systematics, and evolutionary studies.

## Graphical abstract

This study reports the first complete chloroplast genome of *Nepeta griffithii* and compares it with nine previously published *Nepeta plastomes*. Comparative analyses revealed conserved plastome structure, gene content, inverted repeat boundaries, codon usage, and SSR distribution across the genus, while identifying highly variable loci for future phylogenetic and population‐level studies. Phylogenomic analysis placed *N. griffithii* as sister to *N. nuda* , providing new insight into the evolutionary relationships within *Nepeta*.

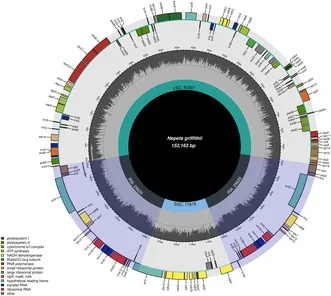

## 1 Introduction

*Nepeta* L. is a large genus of aromatic herbs in the family Lamiaceae, subfamily Nepetoideae, tribe Mentheae, subtribe Nepetinae, comprising approximately 300 accepted species distributed across temperate regions of Europe, Asia, and Africa (Harley et al. 2004; POWO 2026). The greatest species diversity occurs in southwestern Asia (Turkey and Iran) and the western Himalaya including Afghanistan and Pakistan (Asgarpanah et al. 2014; Sharma et al. 2021). The genus is not monophyletic as traditionally circumscribed (Jamzad et al. 2003; Rose et al. 2023), a question that remains unresolved owing to limited molecular sampling. Many species are valued in traditional medicine for their diverse pharmacological activities linked to bioactive compounds such as nepetalactones, 1,8‐cineole, rosmarinic acid, and flavonoids (Formisano et al. 2011; Sharma et al. 2021), underscoring the need for reliable species identification frameworks. *Nepeta griffithii* Hedge is a perennial herb native to Afghanistan and northwestern Pakistan (Hedge 1968; POWO 2026), characterized by branched stems (30–50 cm), broadly ovate leaves grayish white beneath, congested many‐flowered cymes, and light purple corollas (Hedge 1968). Despite its occurrence in a region recognized as a center of *Nepeta* diversity, *N. griffithii* has received limited molecular attention, and to our knowledge no complete chloroplast (cp) genome has been reported for this species. Although the accumulation of Lamiaceae cp genomes has accelerated in recent years, comparative analyses across closely related species within the same genus remain scarce for *Nepeta*, limiting our understanding of intrageneric cp genome evolution and the dynamics of inverted repeat boundaries at fine taxonomic scales.

The cp genome of angiosperms typically displays a conserved quadripartite organization comprising a large single‐copy (LSC) region and a small single‐copy (SSC) region separated by a pair of inverted repeats, IRa and IRb (Daniell et al. 2016). Its predominantly uniparental inheritance, low recombination rate, and moderate substitution rate make the cp genome an informative marker for reconstructing plant phylogeny, assessing population‐level genetic diversity, developing DNA barcodes, and supporting species identification and conservation planning (Daniell et al. 2016; Gitzendanner et al. 2018). Within Nepetinae, cp genome resources have begun to accumulate, with complete cp genomes reported for *Nepeta cataria* (Zhou et al. 2020), *N. hemsleyana* (Bautista et al. 2022), and *N. bracteata* (Chen et al. 2024), among others. More recently, comparative analysis of the *N. nuda* cp genome highlighted signatures of positive selection across several functional genes (e.g., *matK*, *ycf1*, and *rpoC1*) and pinpointed candidate hypervariable barcode loci, including *ycf1* and *rpl32‐trnL‐UAG*, for infrageneric species discrimination (Hejazi and Soorni 2026). However, complete cp genomes are currently available for only 10 *Nepeta* species in the NCBI database, and sampling remains geographically biased, with western Himalayan and Afghan–Pakistani species unrepresented. Given the taxonomic complexity and unresolved phylogenetic relationships within *Nepeta*, expanded cp genome sampling, particularly from under‐collected regions such as the western Himalaya, is essential for robustly resolving species boundaries and clarifying interspecific relationships across the genus.

To address this gap, we sequenced and de novo assembled the complete cp genome of *N. griffithii*. Specifically, we (i) characterized the cp genome of *N. griffithii*, (ii) compared it with nine previously published *Nepeta* cp genomes to elucidate the molecular evolution of the cp genome in *Nepeta*, (iii) identified polymorphic loci suitable for DNA barcoding and phylogenetic studies, and (iv) reconstructed a cp genome‐based phylogeny of *Nepeta* to assess the phylogenetic placement of *N. griffithii* and evaluate interspecific relationships among the sampled species, offering preliminary insights into the phylogenetic coherence of the genus. This study provides the first cp genomic resource for *N. griffithii* and improves the taxonomic and geographic sampling of *Nepeta* cp genomes, thereby supporting future work on the evolution, systematics, and conservation of the genus.

## 2 Materials and Methods

### 2.1 Plant Material, DNA Extraction, and Sequencing

Fresh leaf tissue of *Nepeta griffithii* was collected from the National Agriculture Research Centre, Islamabad, Pakistan (coordinates: 33.671482, 73.126396) in April 2017. The plant was identified by Dr. Sayed Afzal Shah, and a voucher specimen under accession number RAW100892 was deposited at the National Herbarium of Pakistan, maintained by the Pakistan Agriculture Research Centre. The collected leaves were promptly desiccated in silica gel to maintain DNA integrity. Total genomic DNA was extracted from approximately 20 mg of the dried material using a Plant Genomic DNA Kit (TIANGEN Biotech, Beijing, China), following the manufacturer's protocol. DNA concentration and purity were assessed with a Qubit 4.0 fluorometer (Thermo Fisher Scientific, USA), and DNA integrity was evaluated by electrophoresis on a 1% agarose gel. A paired‐end sequencing library with an insert size of ~350 bp was constructed and sequenced paired end (2 × 150 bp) on DNBSeq at Annoroad (Beijing, China), yielding whole‐genome short‐read data suitable for organelle assembly.

### 2.2 De Novo Assembly and Annotation

The raw reads of *N. griffithii* were processed using the Chloroplast Genome Analysis Suite (CGAS v1.0.1). Module 1 of this pipeline was used for quality trimming with fastp (Chen 2025) followed by de novo assembly via GetOrganelle v1.7.7 (Jin et al. 2020), employing embryophyte cp genome seed sequences and a series of k‐mer values (21, 45, 65, 85, and 105). The circularity of the assembled contig was verified, and the average sequencing depth was calculated by mapping the cleaned reads back to the assembly using BWA‐MEM (Li 2013) and SAMtools (Danecek et al. 2021). The coverage map was generated in Geneious Prime 2024 (Biomatters, New Zealand).

Gene annotation was performed using GeSeq (Tillich et al. 2017), with tRNA boundaries further validated by tRNAscan‐SE v2.0 (Chan and Lowe 2019) and ARAGORN v1.2 (Laslett and Canback 2004). In parallel, CGAS Module 2 was used to execute PGA (Qu et al. 2019) using designated *Nepeta* reference genomes (OQ689450, MT663220, OP186463). The outputs from both annotation streams were cross‐verified and manually curated in Geneious Prime 2024 (Biomatters) to resolve any discrepancies in start/stop codons, intron‐exon boundaries, and IR junction pseudogenes. Circular genome maps were visualized with Chloroplot (Zheng et al. 2020). The GenBank submission file was generated using CGAS. The finalized *N. griffithii* cp genome was submitted to GenBank under accession number PZ342510.

### 2.3 Comparative Cp Genome Analysis

The complete cp genome sequences of nine additional *Nepeta* species (*N. bracteata*, *N. cataria* , *N. dentata* , *N. hemsleyana*, *N. nuda* , *N. santoana*, *N. stewartiana*, *N. tenuifolia*, and *N. thomsonii*) were retrieved from the NCBI GenBank database (Table 1). All 10 *Nepeta* cp genomes were compared. The LSC, SSC, and IR lengths, overall and regional guanine‐cytosine (GC) content, gene inventories, and intron dimensions were calculated using CGAS v1.0.1 (Abdullah, Yan, and Tian 2026). The dynamics of the inverted repeat (IR) boundaries, specifically the LSC/IRb, IRb/SSC, SSC/IRa, and IRa/LSC junctions, were inspected for expansion or contraction events using CPJSdraw v0.0.1 (Li et al. 2023).

**TABLE 1:** Chloroplast genome features of *Nepeta* species.

Species	Genome length (bp)				GC (%)				GC (%)			Accession number
	Complete	LSC	SSC	IR	Complete	LSC	SSC	IR	tRNA	rRNA	CDS	
*Nepeta bracteata*	151,588	82,819	17,557	25,606	37.91	35.98	31.84	43.10	52.97	55.21	38.06	OQ689450
*Nepeta cataria*	152,339	83,340	17,605	25,697	37.85	35.90	31.65	43.20	53.01	55.30	37.98	MT663220
*Nepeta dentata*	151,893	83,258	17,407	25,614	37.95	36.02	32.08	43.07	53.01	55.19	38.10	OP186463
*Nepeta griffithii*	152,163	83,307	17,616	25,620	37.84	35.85	31.95	43.09	53.12	55.21	38.04	PZ342510
*Nepeta hemsleyana*	152,171	83,214	17,605	25,676	37.86	35.93	31.87	43.04	53.04	55.19	38.07	MZ618620
*Nepeta nuda*	152,194	83,380	17,584	25,615	37.85	35.90	31.88	43.08	52.90	55.21	38.07	PV930134
*Nepeta santoana*	151,823	83,023	17,616	25,592	37.84	35.87	31.89	43.09	52.94	55.19	38.04	PV837617
*Nepeta stewartiana*	152,348	83,455	17,631	25,631	37.87	35.91	32.00	43.07	53.01	55.21	38.06	MT733874
*Nepeta tenuifolia*	151,254	82,663	17,339	25,626	37.85	35.88	31.78	43.09	52.97	55.21	38.02	NC053707
*Nepeta thomsonii*	152,210	83,302	17,572	25,668	37.88	35.93	32.01	43.04	53.01	55.21	38.04	OP186466

*Note:* Complete: complete chloroplast genome; CDS: protein‐coding gene.

Abbreviations: GC, guanine‐cytosine content; IR, inverted repeat region; LSC, large single‐copy region; rRNA, ribosomal RNA; SSC, small single‐copy region; tRNA, transfer RNA.

CGAS was also used to calculate relative synonymous codon usage (RSCU) values and amino acid frequencies across the protein‐coding genes of all 10 taxa. Simple sequence repeats (SSRs) were identified using CGAS v1.0.1, applying detection thresholds of ≥ 10 repeat units for mononucleotides, ≥ 5 for dinucleotides, ≥ 4 for trinucleotides, and ≥ 3 for tetra‐, penta‐, and hexanucleotide motifs. The distribution of SSRs across genic, intronic, and intergenic compartments was recorded.

### 2.4 Nucleotide Diversity, Candidate Polymorphic Loci, and Phylogenetic Reconstruction

Nucleotide diversity (*π*) was estimated for all coding and non‐coding regions of the 10 *Nepeta* cp genomes using CGAS v1.0.1 Module 13 (Abdullah, Yan, and Tian 2026) to detect highly polymorphic loci with potential application in DNA barcoding and phylogenetic studies.

Phylogenetic relationships were inferred from a concatenated dataset of 79 shared protein‐coding genes extracted from the 10 *Nepeta* taxa and the outgroup *Glechoma hederacea* (PP203125). Gene extraction, alignment, concatenation into a supermatrix, and partition file generation were carried out using CGAS. The nucleotide sequences of the extracted genes were aligned independently using MAFFT v7 (Katoh and Standley 2013) through the CGAS alignment module. Maximum likelihood (ML) tree construction was performed in IQ‐TREE v3.0.1 (Wong et al. 2026) under a partitioned scheme. The best‐fitting nucleotide substitution model for each gene partition was determined through ModelFinder based on the Bayesian Information Criterion (BIC) (Kalyaanamoorthy et al. 2017). Branch reliability was assessed using 1000 ultrafast bootstrap (UFBoot) replicates with the‐bnni option to reduce the risk of overestimating branch support (Hoang et al. 2018) alongside 1000 SH‐aLRT iterations. The resulting phylogenetic tree was annotated and visualized using the Interactive Tree of Life (iTOL) web server (Letunic and Bork 2024).

## 3 Results

### 3.1 Comparative Analysis of Cp Genome Features

The complete cp genomes of all 10 *Nepeta* species exhibited the typical quadripartite structure, comprising a large single‐copy (LSC) region, a small single‐copy (SSC) region, and two IR regions, as illustrated by the newly assembled *N. griffithii* cp genome (Figure 1). High‐throughput sequencing produced approximately 54.46 million raw reads (54,457,230), which yielded ~54.46 million clean reads (54,456,216; 8.14 Gb) following quality control filtering, demonstrating high sequencing accuracy with a Q20 rate of 98.89% and a Q30 rate of 97.95%. The assembly of the newly sequenced *N. griffithii* cp genome was supported by high read coverage, with 501,653 mapped reads specifically aligning to the plastome and an average sequencing depth of 494.1× (Figure S1). Among the studied species, the complete cp genome lengths ranged from 151,254 bp in *N. tenuifolia* to 152,348 bp in *N. stewartiana*, with an average length of approximately 151,998 bp (Table 1). Specifically, the LSC region ranged from 82,663 bp (*N. tenuifolia*) to 83,455 bp (*N. stewartiana*), while the SSC region varied from 17,339 to 17,631 bp. The IR regions showed minor variations, ranging from 25,592 bp (*N. santoana*) to 25,697 bp ( *N. cataria* ), with an average length of 25,635 bp.

**FIGURE 1:**
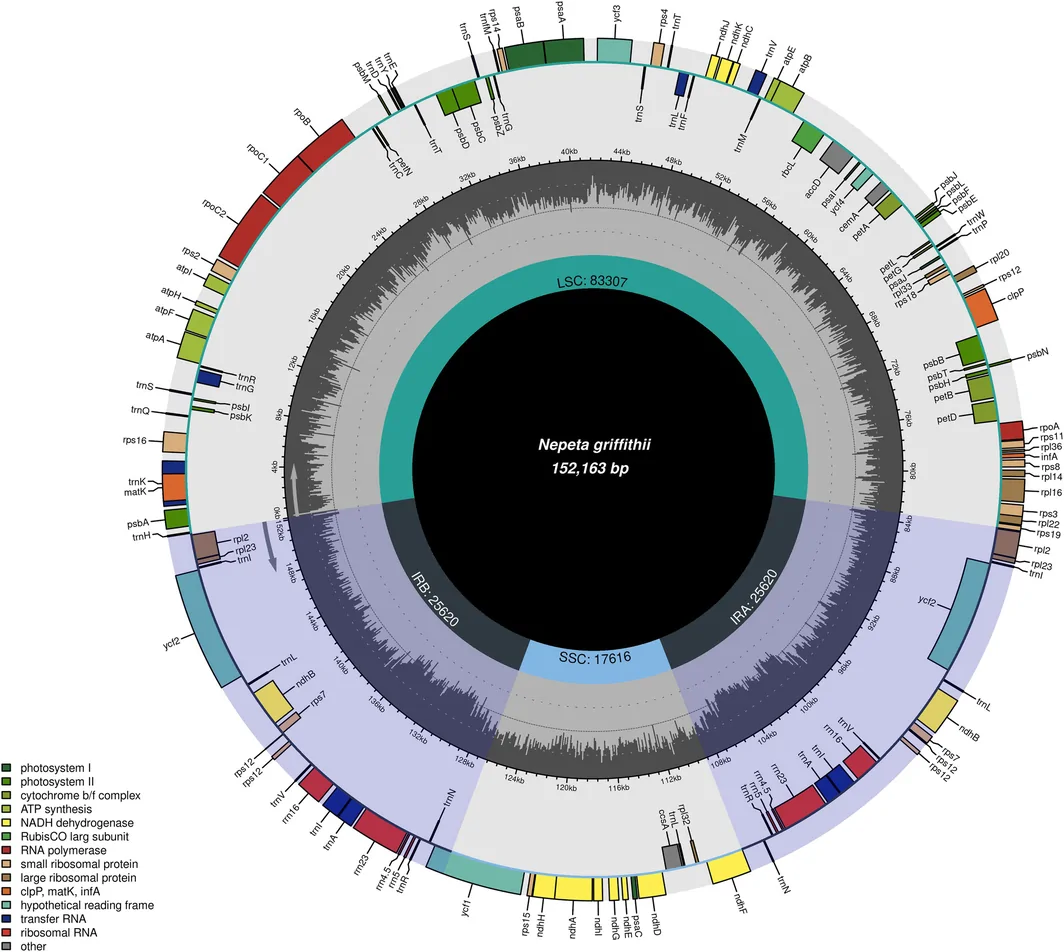
Circular map of the complete chloroplast genome of *N. griffithii*. Genes are colored by functional category. Genes shown on opposite sides of the circle are transcribed in opposite directions. The inner plot shows GC content variation across the cp genome, and the LSC, SSC, IRa, and IRb regions are indicated.

Gene content was also highly conserved across the 10 *Nepeta* species. Each complete cp genome contained 113 unique genes, comprising 79 protein‐coding genes (CDS), 30 transfer RNA (tRNA) genes, and 4 ribosomal RNA (rRNA) genes (Table 2; Table S1). The inverted repeat (IR) regions duplicated a total of 16 genes: 5 protein‐coding genes (*ndhB*, *rpl2*, *rpl23*, *rps7*, *ycf2*), 7 tRNA genes (*trnA‐UGC*, *trnI‐CAU*, *trnI‐GAU*, *trnL‐CAA*, *trnN‐GUU*, *trnR‐ACG*, *trnV‐GAC*), and all 4 rRNA genes (*rrn4.5*, *rrn5*, *rrn16*, *rrn23*). Notably, no pseudogenes were identified in any of the analyzed genomes, indicating complete gene conservation within the genus.

**TABLE 2:** Functional classification of genes in the chloroplast genome of *Nepeta* species.

Category for genes	Group of genes	Name of genes	Total
Self‐replication	Large subunit of ribosome	*rpl14*, rpl16*, rpl2* ^*a*^, *rpl20*, *rpl22*, *rpl23* ^*a*^, *rpl32*, *rpl33*, *rpl36*	11
	Small subunit of ribosome	*rps11*, rps12**, *rps14*, *rps15*, rps16*, *rps18*, *rps19*, *rps2*, *rps3*, *rps4*, *rps7* ^*a*^, *rps8*	13
	DNA dependent RNA polymerase	*rpoA*, *rpoB*, rpoC1*, *rpoC2*	4
	rRNA genes	*rrn16* ^*a*^, *rrn23* ^*a*^, *rrn4.5* ^*a*^, *rrn5* ^*a*^	8
	tRNA genes	trnA‐UGC* ^*a*^, *trnC‐GCA*, *trnD‐GUC*, *trnE‐UUC*, *trnF‐GAA*, *trnG‐GCC*, trnG‐UCC*, *trnH‐GUG*, *trnI‐CAU* ^*a*^, trnI‐GAU* ^*a*^, trnK‐UUU*, *trnL‐CAA* ^*a*^, trnL‐UAA*, *trnL‐UAG*, *trnM‐CAU*, *trnN‐GUU* ^*a*^, *trnP‐UGG*, *trnQ‐UUG*, *trnR‐ACG* ^*a*^, *trnR‐UCU*, *trnS‐GCU*, *trnS‐GGA*, *trnS‐UGA*, *trnT‐GGU*, *trnT‐UGU*, *trnV‐GAC* ^*a*^, trnV‐UAC*, *trnW‐CCA*, *trnY‐GUA*, *trnfM‐CAU*	37
Photosynthesis	Photosystem I	*psaA*, *psaB*, *psaC*, *psaI*, *psaJ*	5
	Photosystem II	*psbA*, *psbB*, *psbC*, *psbD*, *psbE*, *psbF*, *psbH*, *psbI*, *psbJ*, *psbK*, *psbL*, *psbM*, *psbN (pbf1)*, *psbT*, *psbZ (lhbA)*	15
	NADPH dehydrogenase	ndhA*, ndhB* ^*a*^, *ndhC*, *ndhD*, *ndhE*, *ndhF*, *ndhG*, *ndhH*, *ndhI*, *ndhJ*, *ndhK*	12
	Cytochrome b/f complex	*petA*, petB*, petD*, *petG*, *petL*, *petN*	6
	Subunits of ATP synthase	*atpA*, *atpB*, *atpE*, atpF*, *atpH*, *atpI*	6
	Large subunit of Rubisco	*rbcL*	1
	Photosynthesis assembly genes	ycf3 (pafI)**, *ycf4 (pafII)*	2
Other genes	Protease	clpP**	1
	Maturase	*matK*	1
	Envelope membrane protein	*cemA*	1
	Subunit of Acetyl‐CoA‐carboxylase	*accD*	1
	C‐type cytochrome synthesis gene	*ccsA*	1
	Translation initiation factor	*infA*	1
	Conserved open reading frames	*ycf1* ^*a*^, *ycf2* ^*a*^	3
		Total number of genes	129

*Note:* * and ** indicate genes containing one and two introns, respectively; Superscript “a” indicates duplicated genes in inverted repeat (IR) regions. The *rps12* gene is a trans‐spliced gene and is not marked as duplicated despite appearing in multiple locations.

### 3.2 GC Content Distribution

The cp genomes exhibited remarkably consistent overall GC content, ranging from 37.84% to 37.95% (Table 1). However, regional differences were observed across the quadripartite structure. The IR regions displayed significantly higher GC content (43.04%–43.20%) compared to the LSC (35.85%–36.02%) and SSC (31.65%–32.08%) regions. This pattern of GC enrichment in the IRs is consistent with the presence of GC‐rich rRNA genes.

### 3.3 Intron Content and Length Variation

The gene and intron content remained highly conserved across the 10 *Nepeta* species. Analysis identified 18 intron‐containing genes, including 12 protein‐coding genes and six tRNA genes, with conserved positions across species (Figure 2; Table S2). Among the protein‐coding genes, *ycf3* and *clpP* contained two introns each, while *rps12* was identified as a trans‐splicing gene with its 5′ exon located in the LSC region and the 3′ exon duplicated in both IR regions.

**FIGURE 2:**
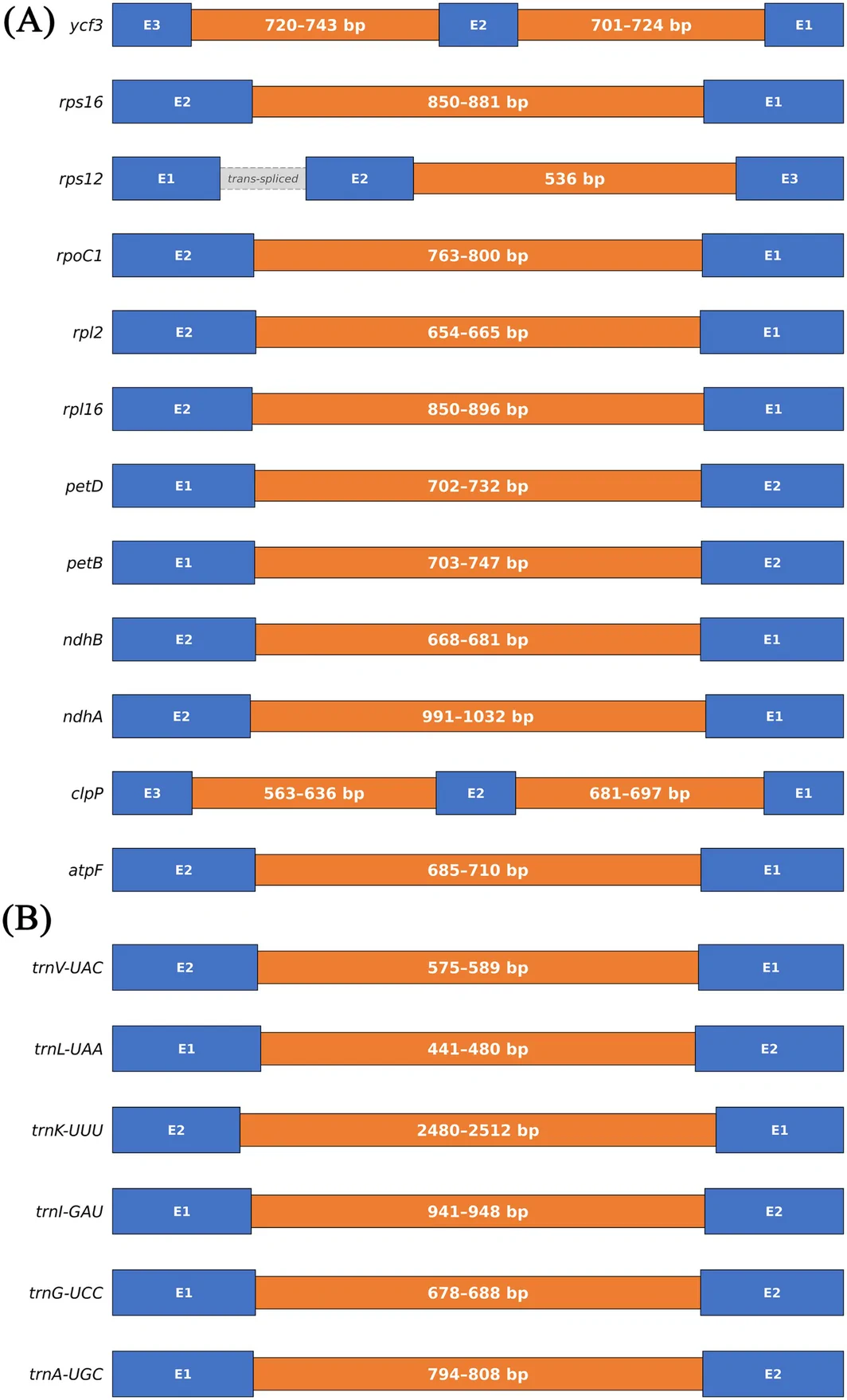
Intron organization of chloroplast genes in the 10 *Nepeta* species. (A) Protein‐coding genes contain one or two introns. (B) tRNA genes contain a single intron. Exons are shown as blue blocks, and introns are shown as orange blocks. Numbers indicate intron‐length ranges in base pairs across the sampled cp genomes. The trans‐spliced structure of *rps12* is indicated.

For tRNA genes, the longest intron was observed in *trnK‐UUU* (2480–2512 bp), with the *matK* gene located within this intron, while the shortest intron was found in *trnL‐UAA* (441–480 bp). Among protein‐coding gene introns, *ndhA* exhibited the longest intron (991–1032 bp), whereas the cis‐spliced intron of *rps12* was the shortest and was perfectly conserved at 536 bp across all species. Intron lengths were generally conserved, although the first intron of *clpP* showed the highest length variation (563–636 bp), followed by *rpl16* (850–896 bp).

### 3.4 Inverted Repeat Contraction and Expansion

Comparative analysis of the IR boundaries among the 10 *Nepeta* cp genomes revealed highly conserved junction patterns with only minor variation in boundary positions (Figure 3). At the LSC/IRb junction (JLB), the *rps19* gene consistently spanned the boundary, with 217–238 bp located in the LSC region and 41–62 bp extending into the IRb region. The *rpl2* gene, duplicated in the IR regions, is located near JLB in IRb and near JLA in IRa, with a length of 1479–1490 bp per copy. At the IRb/SSC junction (JSB), *ndhF* crossed the boundary, with 32–76 bp in IRb and 2156–2200 bp in the SSC region. At the SSC/IRa junction (JSA), *ycf1* crossed the boundary, with 4457–4497 bp located in the SSC region and 1056–1100 bp extending into the IRa region. At the IRa/LSC junction (JLA), *trnH* was consistently located in the LSC region, 5–13 bp from the boundary. Overall, the IR boundary architecture was highly conserved across the 10 *Nepeta* species, suggesting that only limited IR expansion and contraction occurred during cp genome evolution in this genus.

**FIGURE 3:**
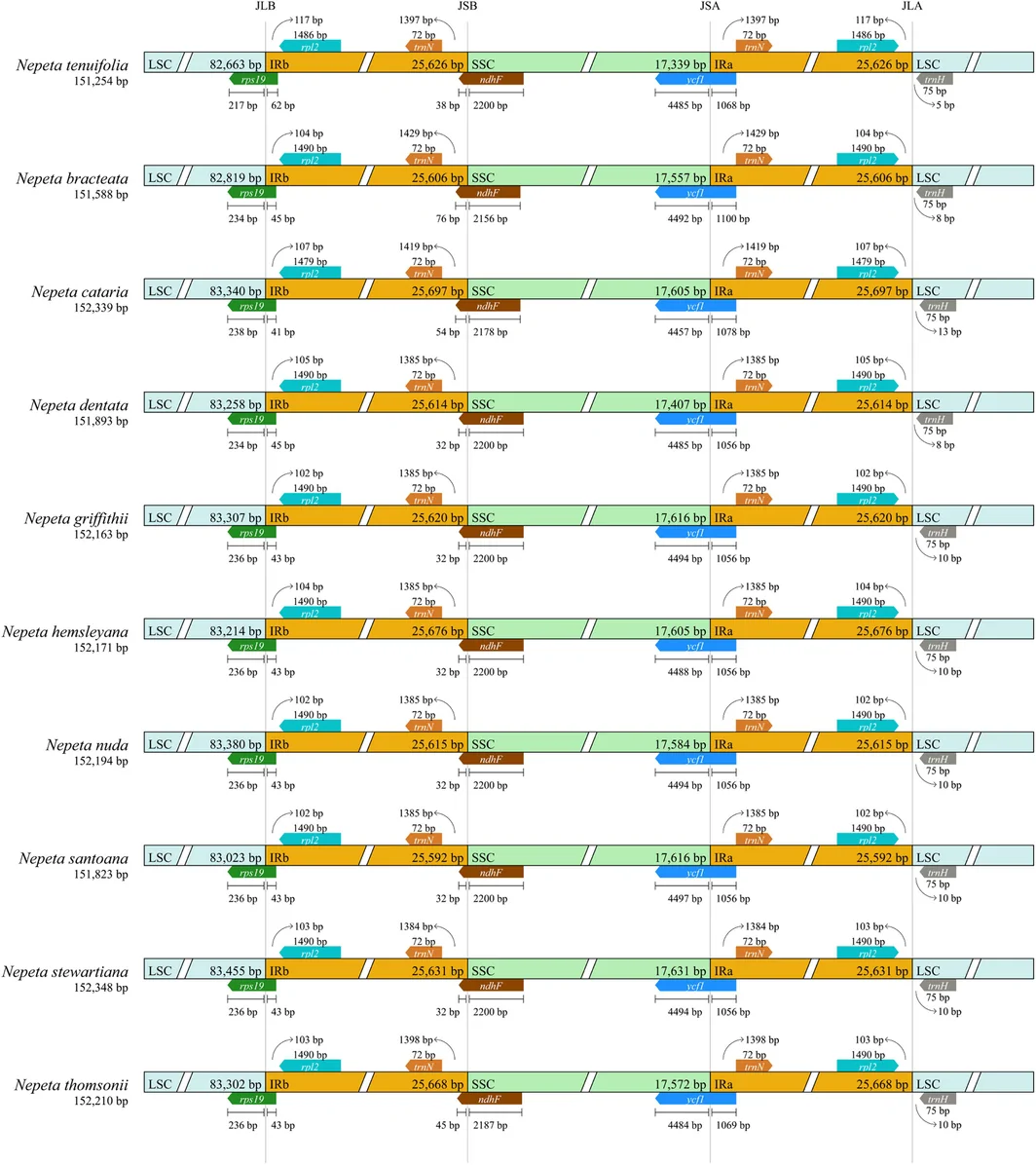
Comparison of IR boundary regions among the 10 *Nepeta* chloroplast genomes. The four junctions, JLB, JSB, JSA, and JLA, are shown for each species. Genes near the junctions are displayed as colored blocks, and numbers indicate gene‐boundary distances or fragment lengths in base pairs.

### 3.5 Codon Usage and Amino Acid Composition

The 10 *Nepeta* species showed highly similar codon usage patterns and amino acid frequencies. Analysis of relative synonymous codon usage (RSCU) showed that most amino acids were encoded by codons ending in A or T at the third position, with RSCU values generally > 1 indicating preference (Figure 4A; Table S3). Among all amino acids, leucine was the most frequently encoded (10.70%–10.75%), followed by isoleucine (8.49%–8.57%), serine (7.81%–7.95%), and glycine (6.74%–6.80%) (Figure 4B; Table S4). In contrast, cysteine was the least frequently encoded (1.11%–1.16%), followed by tryptophan (1.73%–1.76%) and methionine (2.32%–2.39%).

**FIGURE 4:**
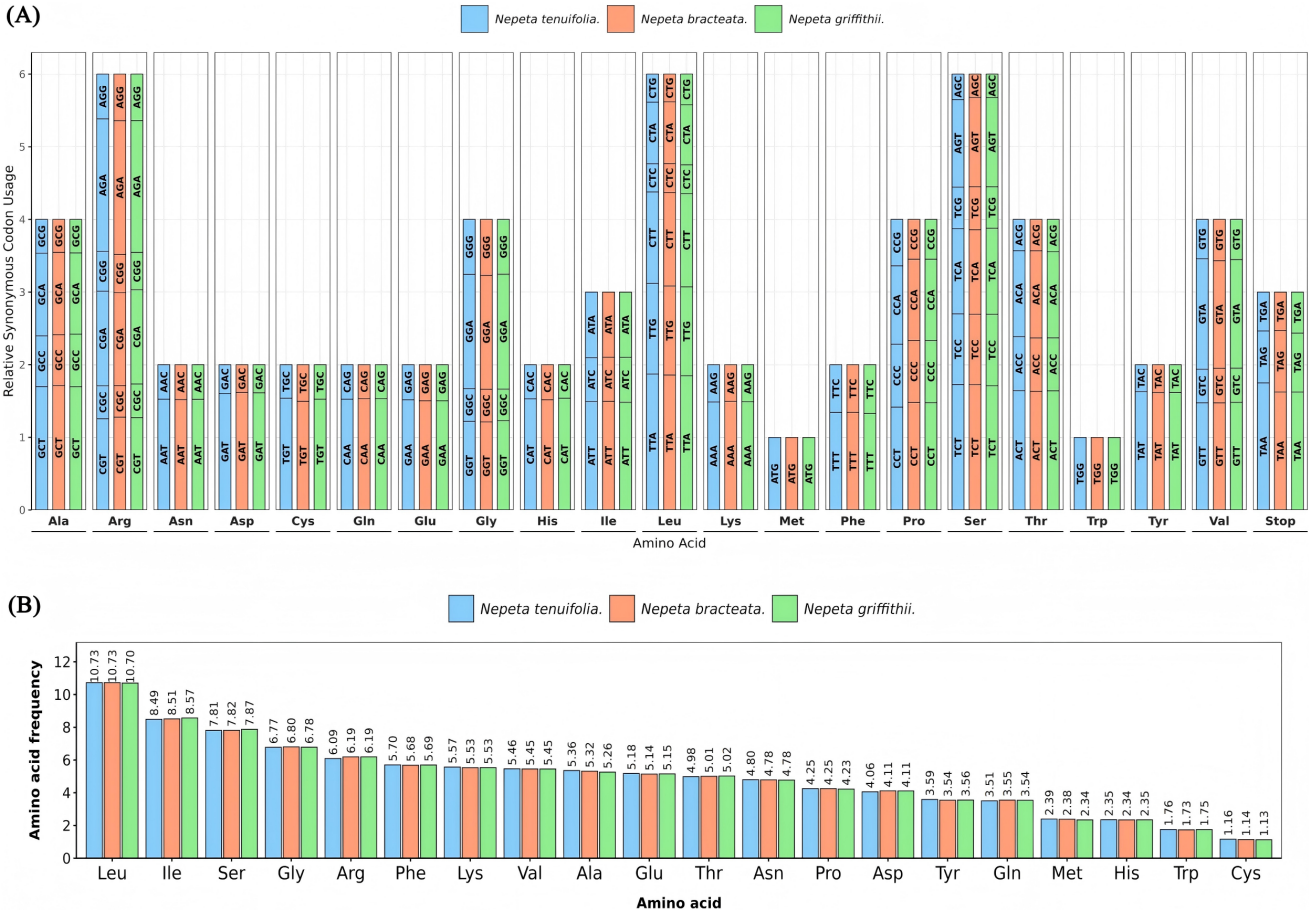
Codon usage and amino acid composition in selected *Nepeta* chloroplast genomes. (A) Relative synonymous codon usage (RSCU) values grouped by amino acid. (B) Amino acid frequencies in chloroplast protein‐coding genes.

### 3.6 Simple Sequence Repeat (SSR) Analysis

Simple sequence repeat (SSR) analysis revealed a total of 387 SSRs across the 10 species, with individual genomes containing between 31 and 51 SSRs (Figure 5; Table S5). Mononucleotide SSRs were the most abundant (46.2%–72.7%), followed by dinucleotide (9.8%–25.6%) and tetranucleotide (9.8%–22.2%) repeats. Among mononucleotide SSRs, A/T repeats dominated overwhelmingly, consistent with the AT‐rich nature of cp genomes, while AT/TA motifs were the most common among dinucleotide SSRs. The majority of SSRs were located in intergenic spacer (IGS) regions (61.3%–74.4%), with smaller proportions found in genic regions (15.7%–22.2%) and introns (7.7%–19.4%).

**FIGURE 5:**
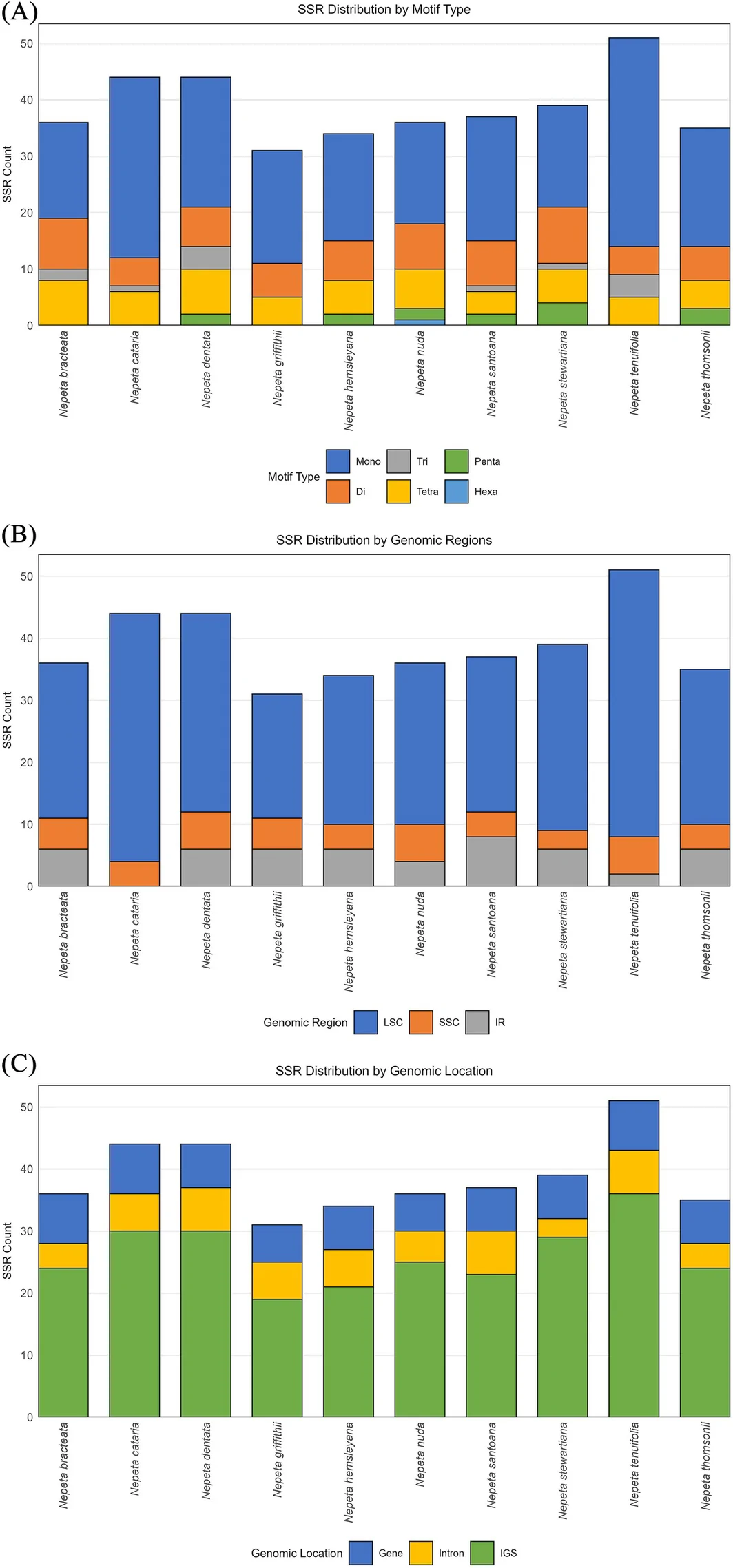
Distribution of simple sequence repeats (SSRs) in the 10 *Nepeta* chloroplast genomes. (A) SSRs classified by motif type. (B) SSR distribution across LSC, SSC, and IR regions. (C) SSR distribution across genic regions, introns, and intergenic spacers.

### 3.7 Nucleotide Diversity and Polymorphic Loci

Intergenic spacer regions, intronic regions, and genic regions were analyzed to identify highly variable regions across all sampled species. The overall average nucleotide diversity (*π*) across 236 analyzed cp genome regions was 0.022. Intergenic spacer regions exhibited the highest average diversity (*π* = 0.035), followed by intronic regions (*π* = 0.021) and genic regions (*π* = 0.011) (Figure 6).

**FIGURE 6:**
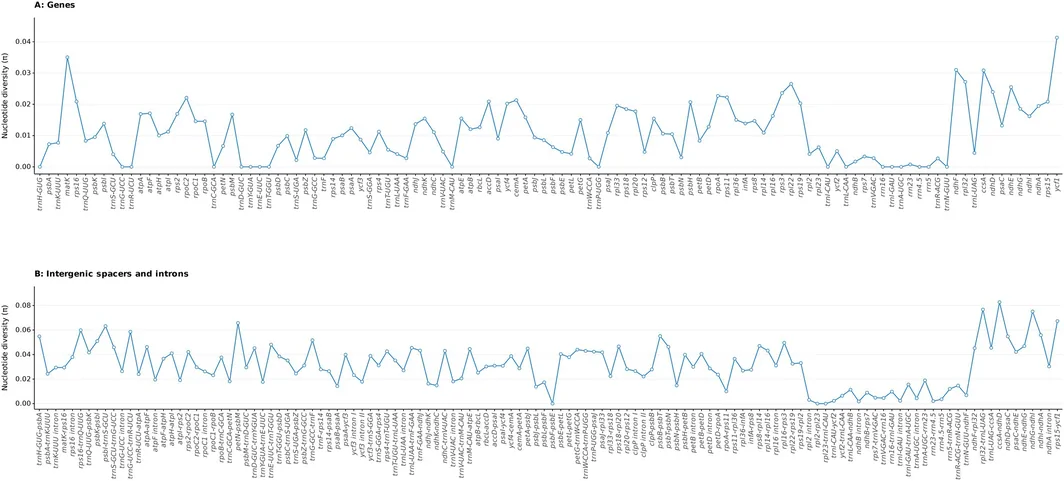
Nucleotide diversity (*π*) across chloroplast genomic regions in the 10 *Nepeta* species. (A) Nucleotide diversity values for genic regions. (B) Nucleotide diversity values for intergenic spacers and introns. Each point represents one annotated region.

Several highly polymorphic loci were identified as potential candidates for phylogenetic and population‐level studies. The most polymorphic intergenic spacer was *ccsA‐ndhD* (*π* = 0.083), followed by *rpl32‐trnL‐UAG* (*π* = 0.077) and *ndhG‐ndhI* (*π* = 0.075). Among protein‐coding sequences, *ycf1* exhibited the highest diversity (*π* = 0.041), whereas the commonly used DNA barcode *rbcL* showed relatively low diversity (*π* = 0.013).

### 3.8 Phylogenetic Analysis

The maximum likelihood phylogeny inferred from the concatenated dataset of 79 shared protein‐coding genes recovered all nodes with full bootstrap support (UFBoot = 100%) (Figure 7). Within the ingroup, *Nepeta tenuifolia* was resolved as sister to all remaining *Nepeta* species, followed by *N. cataria* , which occupied the second‐earliest diverging position and exhibited the longest branch among the sampled taxa. *Nepeta dentata* diverged next, sister to a clade of seven species. Within this clade, *N. santoana* was sister to a core group of six species in which *N. hemsleyana* was sister to a clade comprising two subclades: *N. griffithii* + *N. nuda* and *N. bracteata* + (*N. stewartiana* + *N. thomsonii*). The sister relationship between *N. stewartiana* and *N. thomsonii* was characterized by very short branch lengths, indicating minimal sequence divergence between these two species. The newly sequenced *N. griffithii* was placed as sister to *N. nuda* with strong support, and this pair was in turn sister to the *N. bracteata*–*N. stewartiana*–*N. thomsonii* clade.

**FIGURE 7:**
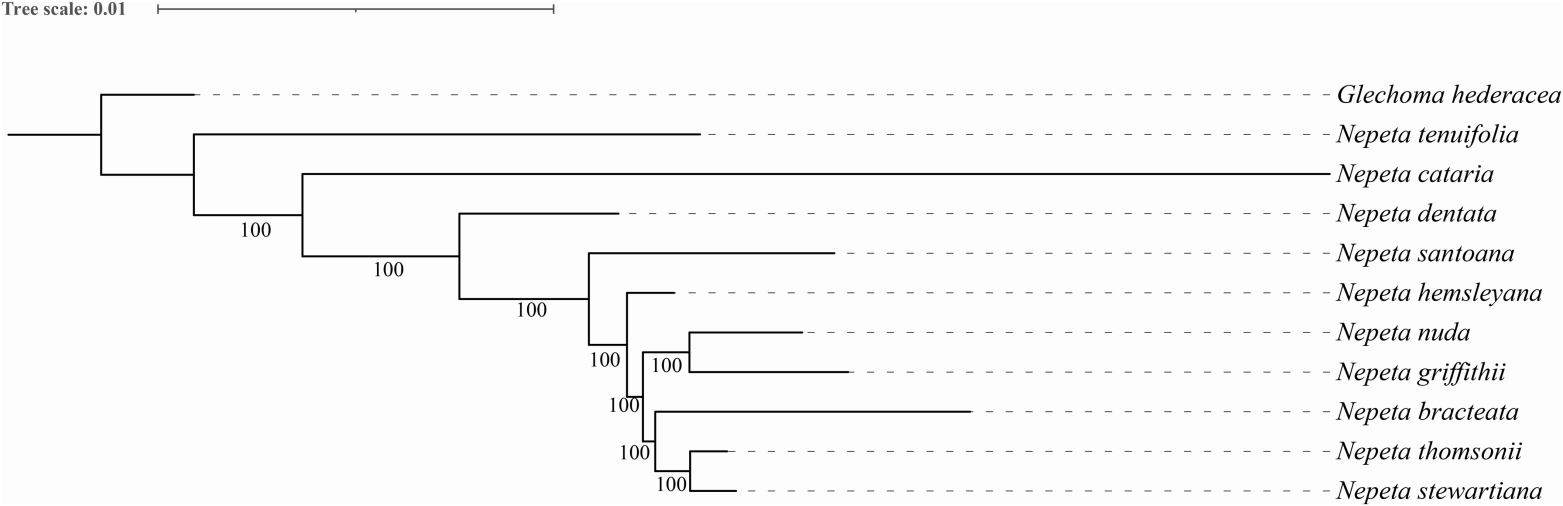
Maximum likelihood phylogeny of *Nepeta* based on 79 chloroplast protein‐coding genes. The tree was reconstructed using IQ‐TREE with *Glechoma hederacea* (PP203125) as the outgroup. Node labels indicate branch‐support values, and the scale bar represents substitutions per site.

## 4 Discussion

This study presents the first comprehensive comparative analysis of cp genomes across 10 *Nepeta* species, including the newly sequenced *N. griffithii*. The conserved genome architecture—spanning size, gene order, and gene content—aligns with patterns observed across Lamiaceae (Chen et al. 2024; Wu et al. 2021) and broader angiosperm lineages (Abdullah, Rahmatulla, et al. 2025; Daniell et al. 2016; Wicke et al. 2011), reinforcing the cp genome's role as a slowly evolving genomic compartment in plants. Such evolutionary stasis likely stems from several well‐established mechanisms in plant organelle biology (Wicke et al. 2011): cp genes organized into operons, uniparental inheritance, DNA repair, and rare cp genome fusion events that preserve lineage‐specific integrity.

The conserved GC content profile—with elevated GC in inverted repeats relative to single‐copy regions—mirrors trends observed across diverse plant clades (Chen et al. 2024; Wu et al. 2021; Zhao et al. 2021) and correlates with IR‐localized rRNA genes that are subject to strong purifying selection maintaining ribosomal function. Analysis of IR boundaries also confirmed high conservation, with the four junctions exhibiting gene arrangements consistent with those documented in *Salvia* (Wu et al. 2021). IR boundary fluctuations—known to cause gene duplications and pseudogene origination in other lineages (Abdullah, Haram, et al. 2025; Daniell et al. 2016; Raubeson et al. 2007; Wicke et al. 2011)—showed minimal variation within the genus. This level of IR conservation in *Nepeta* is comparable to that reported for Lamiaceae and other angiosperms (Abdullah, Yan, Yir‐erong, et al. 2026; Chen et al. 2024; Wu et al. 2021), whereas the subfamily Asclepiadoideae (Apocynaceae) exhibits substantially greater IR dynamics, with SSC‐region genes incorporated into the IRs (Abdullah, Haram, et al. 2025), highlighting that IR boundary stability versus dynamism is a lineage‐specific property. Intron content and codon usage patterns were similarly conserved, reinforcing the view that cp introns are subject to strong purifying selection due to their essential roles in RNA splicing and gene expression regulation (Bock 2007).

SSR analysis revealed a predominance of mononucleotide A/T repeats concentrated in intergenic spacer regions, consistent with the AT‐rich nature of cp genomes and with previous reports from Lamiaceae and other angiosperm lineages (Abdullah, Rahmatulla, et al. 2025; Chen et al. 2024; Wu et al. 2021). Because chloroplast SSRs exhibit high polymorphism and inter‐specific variation, they represent valuable molecular tools for assessing intraspecific genetic diversity and population structure (Provan et al. 2001; Xue et al. 2012). For a species such as *N. griffithii*, which is distributed across geographically fragmented habitats in Afghanistan and northwestern Pakistan, the cpSSR markers identified here can inform conservation prioritization by quantifying genetic differentiation among isolated populations.

Nucleotide diversity analysis identified several highly polymorphic loci. Among these, *rpl32‐trnL‐UAG* has been reported as a hypervariable region in *Salvia* (Wu et al. 2021), and *ycf1* consistently ranks among the most variable cp genome coding regions across angiosperms (Dong et al. 2015). Among protein‐coding genes, *ycf1* showed the highest nucleotide diversity (*π*) in our dataset, with elevated values also reported in *Salvia* (Wu et al. 2021) and across angiosperms (Dong et al. 2015), whereas *rbcL* showed low diversity, consistent with its strong functional constraint and predominantly housekeeping role in carbon fixation. These findings are in strong agreement with recent plastome comparisons in *Nepeta* by Hejazi and Soorni (2026), who similarly identified *ycf1* and the *rpl32‐trnL‐UAG* spacer as primary hypervariable regions providing superior species‐level resolution compared to conventional markers like *matK* and *rbcL*. Furthermore, Chen et al. (2024) highlighted *ndhG‐ndhI* as a candidate polymorphic region, which also emerged as one of the most divergent intergenic spacers in our 10‐species analysis (*π* = 0.075). These polymorphic loci represent strong candidate markers for species‐level DNA barcoding and phylogenetic analysis within *Nepeta*. The *ycf1* gene and the *rpl32‐trnL‐UAG* intergenic spacer, both widely validated across angiosperms (Dong et al. 2015; Shaw et al. 2007) and corroborated across independent *Nepeta* plastome datasets (Hejazi and Soorni 2026), are well‐suited for a multi‐locus barcoding framework for the genus.

The maximum likelihood phylogeny based on 79 concatenated protein‐coding genes recovered a fully resolved topology with maximum bootstrap support at all nodes, surpassing the resolution achieved in earlier studies relying on nrITS alone (Jamzad et al. 2003) or a limited number of cp and nuclear loci (Rose et al. 2023), where several backbone nodes within *Nepeta* s.s. remained weakly supported. Within the ingroup, *N. tenuifolia* was recovered as the earliest‐diverging lineage, followed by *N. cataria* , which exhibited the longest branch—indicative of elevated nucleotide substitution rates relative to its congeners. The sister relationship between *N. bracteata* and *N. stewartiana* + *N. thomsonii* corroborates Chen et al. (2024), who reported *N. bracteata* as most closely related to *N. stewartiana* based on a six‐species cp genome comparison. Our expanded sampling further resolves *N. thomsonii* as sister to *N. stewartiana* with minimal sequence divergence, suggesting either recent speciation or incomplete lineage sorting between these taxa. The placement of *N. griffithii* as sister to *N. nuda* provides the first molecular evidence for the phylogenetic affinity of this taxonomically poorly studied Afghan‐Pakistani species, linking it to a Eurasian taxon rather than to geographically proximate Himalayan congeners such as *N. stewartiana* or *N. thomsonii*.

Jamzad et al. (2003) recovered *Nepeta* as monophyletic and recognized five major clades corresponding broadly to sectional groupings based on ITS data. However, with only 10 of the approximately 300 accepted species sampled here, it is premature to draw conclusions regarding the monophyly of the genus or the circumscription of infrageneric groups on the basis of cp data alone. Furthermore, cp genome phylogenies reflect maternal lineage history and may differ from nuclear gene trees due to incomplete lineage sorting or hybridization—phenomena documented within Nepetinae (Rose et al. 2023). The phylogenetic framework established here, while limited in taxonomic breadth, provides a robust cp genome backbone that can be expanded as additional *Nepeta* cp genomes become available, enabling integrative multi‐locus analyses—combining cp and nuclear datasets along with morphological analysis—to resolve the systematics of this species‐rich and taxonomically complex genus.

## 5 Conclusion

This study presents the first complete cp genome of *Nepeta griffithii* and provides a comparative framework across 10 *Nepeta* plastomes. Genome architecture, gene content, GC content, intron features, and IR boundaries were highly conserved across all taxa. SSR and nucleotide diversity analyses identified candidate cpSSR markers and polymorphic loci (*ccsA‐ndhD*, *rpl32‐trnL‐UAG*, *ndhG‐ndhI*, *ycf1*) suitable for population genetics and species identification. The ML phylogeny placed *N. griffithii* as sister to *N. nuda* with full bootstrap support, providing the first molecular evidence for its phylogenetic affinity. Expanded taxon sampling and integrative multi‐locus analyses will be essential to resolve the systematics of this complex genus.

## Author Contributions

**Xinyi Li:** conceptualization (equal), data curation (equal), formal analysis (equal), investigation (equal), writing – original draft (equal). **Abdul Sammad:** conceptualization (equal), formal analysis (equal), investigation (equal). **Sayed Afzal Shah:** data curation (supporting), formal analysis (supporting). **Jiahui Sun:** resources (equal), writing – review and editing (supporting). **Aman Karim:** data curation (supporting). **Bartholomew Yir‐erong:** conceptualization (equal), data curation (equal), writing – review and editing (equal). **Xiaoxuan Tian:** conceptualization (equal), resources (equal), writing – review and editing (equal). **Abdullah:** conceptualization (equal), investigation (equal), writing – original draft (equal), writing – review and editing (equal).

## Funding

This study was supported by the Key Project at Central Government Level: The Ability Establishment of Sustainable Use for Valuable Chinese Medicine Resources (2060302).

## Ethics Statement

The authors have nothing to report.

## Conflicts of Interest

The authors declare no conflicts of interest.

## Supporting information


**Figure S1:** Read coverage of the assembled *Nepeta griffithii* chloroplast genome. Cleaned reads were mapped back to the assembled plastome in Geneious Prime. The coverage profile and mapping statistics are shown across the genome.

**Table S1:** Comparative gene analysis of *Nepeta* species.

**Table S2:** Gene intron size comparison of *Nepeta* species plastomes.

**Table S3:** Codon usage analysis of *Nepeta* species.

**Table S4:** Amino acid analysis of *Nepeta* species.

**Table S5:** Distribution of simple sequence repeats of *Nepeta* species.

## Data Availability

The complete chloroplast genome sequence of *Nepeta griffithii* has been deposited in GenBank under accession number PZ342510. The raw sequencing reads have been deposited in NCBI under BioProject accession number PRJNA1456742.
